# An X-ray computed micro-tomography dataset for oil removal from carbonate porous media

**DOI:** 10.1038/sdata.2019.4

**Published:** 2019-01-29

**Authors:** Tannaz Pak, Nathaly Lopes Archilha, Iara Frangiotti Mantovani, Anderson Camargo Moreira, Ian B. Butler

**Affiliations:** 1 School of Science, Engineering, and Design, Teesside University, Borough Road, Middlesbrough, TS1 3BA, UK; 2 Brazilian Synchrotron Light Laboratory (LNLS), Brazilian Center for Research in Energy and Materials (CNPEM), 13083-970, Campinas, Sao Paulo, Brazil; 3 LMPT - Department of Mechanical Engineering, Federal University of Santa Catarina, 88040-900, Florianópolis, Santa Catarina, Brazil; 4 School of Geosciences, The University of Edinburgh, James Hutton Road, Edinburgh, EH9 3FE, UK

**Keywords:** Environmental sciences, Imaging techniques, Crude oil

## Abstract

This study reveals the pore-scale details of oil mobilisation and recovery from a carbonate rock upon injection of aqueous nanoparticle (NP) suspensions. X-ray computed micro-tomography (μCT), which is a non-destructive imaging technique, was used to acquire a dataset which includes: (i) 3D images of the sample collected at the end of fluid injection steps, and (ii) 2D radiogram series collected during fluid injections. The latter allows monitoring fluid flow dynamics at time resolutions down to a few seconds using a laboratory-based μCT scanner. By making this dataset publicly available we enable (i) new image reconstruction algorithms to be tested on large images, (ii) further development of image segmentation algorithms based on machine learning, and (iii) new models for multi-phase fluid displacements in porous media to be evaluated using images of a dynamic process in a naturally occurring and complex material. This dataset is comprehensive in that it offers a series of images that were captured before/during/and after the immiscible fluid injections.

## Background & Summary

Multi-phase fluid transport in porous media is of great significance for a wide range of applications, including environmental processes and oil/gas production. Capillary action^1^ causes trapping of non-wetting fluids in porous media upon injection of the wetting phase. The wetting fluid spreads over a solid surface, preferentially, in the presence of other non-wetting fluid(s)^2^. The trapping phenomena is well-known, its extent and nature has been evidenced directly using high-resolution and non-destructive imaging techniques such as X-ray computed micro-tomography (μCT). Removal of a trapped non-wetting fluid (e.g. oil) from a porous media (e.g. soil or rock) is conventionally achieved by injection of wetting fluids (e.g. water or a chemical-based solution). Novel technologies including injection of nanoparticle (NP) suspensions (both non-reactive and reactive) have received significant attention in the recent years^3,4^. For the reactive NPs (e.g. zero-valent iron) the idea is to degrade the trapped oil in-situ, while the non-reactive NPs (e.g. silica and alumina) alter the fluid/fluid interfacial tension^5,6^ and/or the rock wettability to aid remobilisation. Laboratory, pilot, and field-scale studies have proven that NPs enhance both in-situ degradation^3,7,8^ and remobilisation of trapped oil phases^4,9–11^. However, the details of oil remobilisation at pore-scale which lead to its macro-scale displacement are not clearly understood.

Direct imaging of multi-phase fluid flow processes within porous media using μCT has identified a number of key pore-scale displacement processes that control the flow in synthetic and natural porous media. These include snap-off^12–14^, Haines jumps^15^, and droplet fragmentation^16^.

The present dataset was acquired using μCT methods. These data provide the first evidence on NP-based oil remobilisation in a carbonate rock which was recently published in Scientific Reports^17^. Here we present the dataset behind this publication which includes 2D and 3D tomographic images of the rock sample (and the fluids it confines) during the fluid flow experiment.

In summary the experiment^17^ is composed of a series of μCT monitored fluid injections in a dolomite rock sample, details of the experiment are explained in the methods section. We used silica NPs at concentrations of 0.06 wt% and 0.12 wt%. The particles, once dispersed in deionised water, decrease the oil/water interfacial tension and hence assist with the remobilisation of the trapped oil droplets. This experiment provides direct evidence of formation of an oil in water emulsion during the NP injection at higher concentration.

Modelling of multi-phase fluid flow in porous media has been, traditionally, performed on pore-network models extracted from 2D/3D images of porous material. For a review of pore-network models, the reader is referred to Joekar-Niasar and Hassanizadeh^18^, and Blunt^19^. More recently, performing flow simulations directly on images (2D and 3D) of simple porous material has become possible without the need to extract pore-network models. For an example direct simulation of two-phase flow on a 2D porous media see Rabbani *et al*.^20^. An important, distinctive advantage of this dataset is that it captures the process of fluid displacement within a naturally occurring carbonate rock that has a heterogeneous pore-structure. Sharing this dataset will assist moving towards modelling of fluid flow within more realistic porous media as opposed to idealised ones such as bead-pack systems.

Acquiring a μCT dataset is time and resource demanding. Running a controlled experiment at such small scale is a challenge, therefore, we see great potential in sharing this dataset so that attention can be paid to modelling the trends observed in this experiment. In addition to evaluation of pore-scale models, these images can be used to aid the design of future experiments.

## Methods

The rock sample (D = 12.8 mm, L = 34.5 mm) was encapsulated in epoxy resin and cut to appropriate dimensions to fit in the flow cell used in this study. The flow cell used here is highly X-ray transparent, for design details see Pak *et al*.^14^. The epoxy-encapsulated core was vacuum saturated with deionised water. The core flooding system was assembled and installed on the rotary table of a laboratory-based μCT machine.

**Figure 1:**
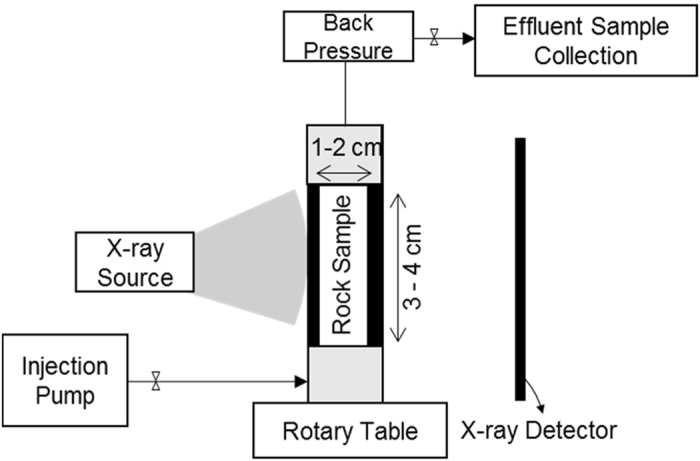
Experimental Set-up used to generate this data set. This set-up is a core flooding system installed on a μCT instrument.

The experimental set-up is shown in Figure 1. The fluid injections were designed to occur under the capillary dominant flow regime (capillary number~10^−7^)^1^ to represent the processes relevant to field-scale operations. A back pressure regulator was used at the outlet end of the core to avoid air bubble generation during the experiments. This back pressure was kept at 518 kPa during the nanofluid injections. Fluid injections were performed from the bottom of the core. For this rock the pore volume (PV) was calculated 850 μL (porosity = 19.1%). The X-ray attenuation of water is negligible compared to the rock (dolomite mineral). Therefore, a 3D scan of the water-saturated rock captures the pore-structure of the rock. From this point the experiment is composed of: (i) fluid injections in the core, (ii) collection of 2D radiograms during the injection steps, and (iii) collection of 3D images at the end of each injection step. The details of the fluids and nanoparticles used in this study is summarised in Table 1.

Oil injection was carried out (more than 10 PVs), subsequent to the initial water saturation, which achieved an initial oil saturation of 78%. Next, deionised water was injected in the core, this represented the waterflooding process that is a common improved oil recovery technique. The subsequent two steps involved injection of nanofluids at 0.06 wt% and 0.12 wt% concentration. After each fluid injection step we closed the valves and started the imaging within 30 min. Table 2 summarises the details of the injections steps.

To increase the X-ray attenuation of the oil phase, 1-iododecane was used as a dopant. The selected oil mixture, therefore, enhanced image contrast while also matching the density of the fluids. The density of 50:50 vol% 1-iododecane/dodecane oil (ρ_o_) is 1.005 g/cm^3^ which is very close to water density (ρ_w_) of 1 g/cm^3^ and that of the two nanofluids (1.0006, 1.0012 g/cm^3^). This eliminated the potential for gravity-driven fluid redistribution during 3D image acquisition.

Three series of 2D radiograms were collected during fluid injections (3000–5000 radiograms, every 2 s). It should be noted that a 2D radiogram of a 3D object represents the cumulative attenuation of the X-ray beam projected on the detector’s plane. The fluid movements within the core were captured on these 2D radiograms. As explained in Pak *et al*. (2018), re-slicing these stacks of radiograms in the Z (time) dimension presents an effective means for direct comparison of fast fluid movements between the different injection steps. 2D radiograms are shared using the .raw format. We note that a .raw image can be automatically converted to a stack of 2D images using imageJ^21^ or other image processing software packages, e.g. Avizo^22^.

The μCT scanner used in this study is a cone-beam system built at the University of Edinburgh. It comprises a 10–160 kV Feinfocus dual transmission and reflection source (reflection used in this study), a Micos UPR-160-air rotary table and a Perkin Elmer 0822 amorphous silicon flat panel X-ray detector with a terbium doped gadolinium oxysulfide scintillator. The collected μCT images captured a field of view of 11 mm side-length, 2–3 mm away from the inlet face of the core. Hence, the images do not show the inlet and outlet of the core. This section was chosen to ensure that capillary end effects do not influence the volume investigated. Each 3D volume is comprised of 2000 radiograms (exposure time: 2 s) captured during a full 360° rotation. A 0.8 mm aluminium filter was used during the scans to reduce the measurement noise and beam hardening effect. The source-sample and sample-detector distances were 37 mm and 549.5 mm, respectively. The X-ray source voltage and current were 120 kV and 195 μA, respectively. The X-ray spot size is 5 μm. Reconstructed 3D images have the voxel resolution of 13.25 μm. The shared 3D images are of 8 bit format. These images were rescaled (after reconstruction in Octopus^23^) with offset and slope of 0 and 7.84 × 10^−3^, respectively.

**Figure 2:**
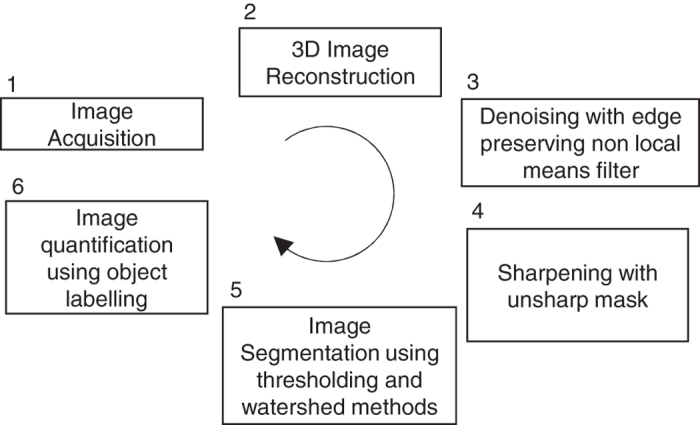
Summary of the post-process and analysis steps taken for the collected 3D images.

Figure 2 summarises the steps taken to post-process and analyse the collected 3D images. We have used the non-local means filter^24^ (applied on XY planes, search window:21, local neighbourhood window: 5, and similarity value: 0.4) followed by the unsharp mask filter^25^ (3D, edge size: 5, and edge contrast: 0.7) both implemented in Avizo^22^ software (version 9).

To ensure our analysis stays consistent within the entire dataset we created a rock mask from the dry rock image using watershed segmentation method^26^. This mask is included with the shared data and is labelled as “Rock-Mask”. Applying this mask on different images within the sequence removed the rock phase which in turn facilitated the segmentation of oil and water phases using simple thresholding. Labelling^27^ was used to identify connected voxels and classify objects in each image.

## Data Records

For each 3D image collected at the end of the injection steps here we share the (i) 2D radiograms, (ii) reconstructed images, (iii) filtered images, and (iv) segmented images (oil and water phases). This dataset also presents three sets of 2D radiograms collected during the injection steps, details of the shared images are summarised in Tables 3, 4, 5 (Data Citation 38).

The dolomite rock of this dataset is a Silurian Dolomite from an outcrop (Thornton formation, located near Chicago, Illinois, in US). This rock has been studied by us since 2011. Readers can find relevant data/images/analysis of this rock in our previous publications including Pak *et al*.^16^, Pak *et al*.^28^, and Pak *et al*.^11^. Additionally, detailed discussions on two-phase flow experiments and wettability (initial and alteration) of this rock in presented in Pak^29^. Data of special interest include:

- Mercury porosimetry (including capillary pressure vs. saturation (Pc-Sat) curves and the extracted pore-throat size distributions)^28^
- Porosity and permeability measurement^30^
- Scanning electron microscopy images^29^
- XRD (X-ray Diffraction) data for this rock examining the rock mineralogy which, in summary, suggests this rock is made of more than 99% dolomite^29^.

**Figure 3:**
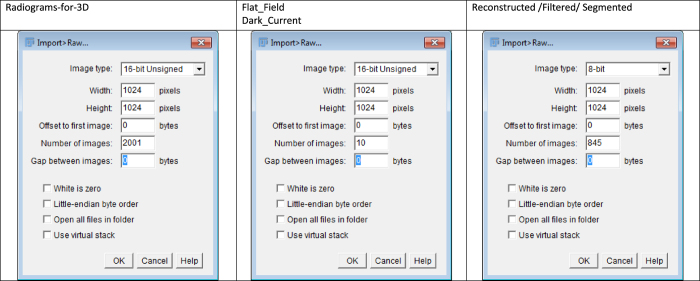
Data viewing Parameters in ImageJ.

**Figure 4:**
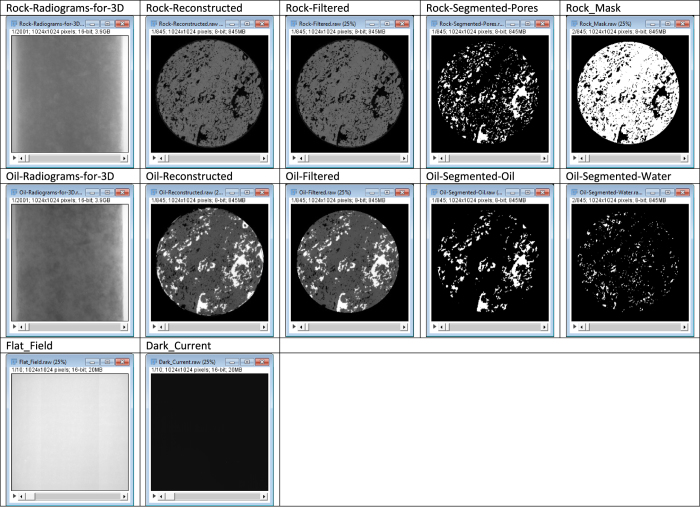
Example slices of the images presented in this dataset.

Table 6 summarises the outputs of this experiment. Figure 3 lists the parameters used for data viewing in ImageJ. Figure 4 shows example slices of the data for reference.

## Technical Validation

**Figure 5:**
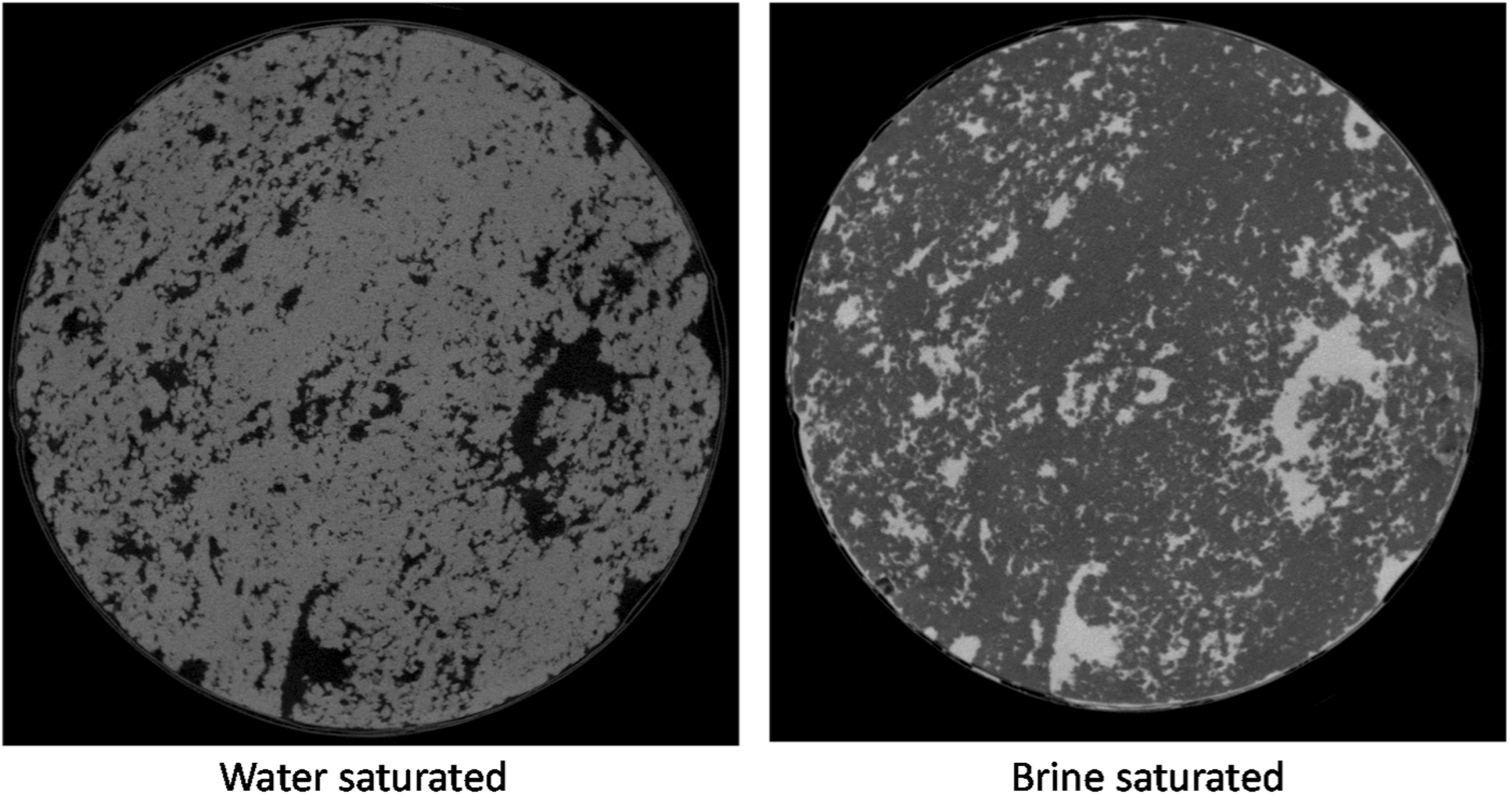
Example μCT slice of the core plug captured after the initial water saturation and KI injection steps, black (water), dark grey (dolomite), and bright grey (brine, 2.5 M KI).

Prior to the injection of oil into the core (i.e. before it becomes a two-phase flow problem), we examined the saturation state of the core for a single fluid phase. The goal was to ensure the initial water saturation was successful, i.e. a system with no trapped air in the rock. A brine phase (KI, 2.5 M) was injected in the core and the sample was scanned. We specifically used the KI salt as iodine is highly X-ray attenuating, this provides a good contrast between the dolomite/brine/potential air phases on the acquired μCT image. Figure 5 shows an example slice of the core. It shows a successful initial saturation which is essential for a reliable image segmentation. In cases were air is present in the core from the beginning image segmentation may result in assigning the air phase to a fluid with close attenuation (here, water due to low contrast). Following this step, the brine was displaced out by water injection (more than ten PVs). Images were post-processed to remove the measurement noise with the aim of producing more reliable segmentations.

The sequences of radiograms collected during the injection steps enabled monitoring of the fluid injections closely, and in particular to assess whether a steady state was being approached or whether fluid mobilisation was still in progress. Also, use of transparent tubing in the injection and production lines allowed us to monitor the injected fluids to ensure no air bubbles were injected in the core. Added to these was the use of back pressure regulator which assisted in running an air-free experiment.

## Usage Notes

Suggested software to process the presented images is ImageJ^21^ (version 1.52 or later) which is an open-source software. In addition to ImageJ we also used Avizo^22^ software (version 9) in our related publication^17^. For filtering the measurement noise we suggest using non-local means^24^ filter, for segmentation we suggest watershed segmentation^26,32^ (implemented both in Avizo^22^ and ImageJ^21^) or WEKA^33^ segmentation (which is a machine learning tool implemented in ImageJ). For 3D image visualisation we suggest using ParaView^34^, Drishti^35^ (both open-source), or Avizo^22^. The 3D reconstructions were made in Octopus 8.5^23^.

The 2D radiograms collected during each 3D scan is of great value for developers of reconstruction algorithms. The 3D reconstructed images are useful for developers of segmentation algorithms and noise filters. In addition, these images can be used to test algorithms developed for artefact correction, e.g. ring artefacts. Although example μCT images can be found in public domain, in most cases smaller and lower resolution images are shared which are not useful in examining the capabilities of algorithms that aim to process larger and higher resolution images. An example platform that enables researchers to share μCT data (and related measurements) of porous material is the “Digital Rock Portal” which was made public in 2015^36^. At this point in time, this platform has close to 20 projects directly related to imaging of dry rocks or fluid flow processes in rocks. Some research groups have made μCT images available through their university webpages such as the data shared by the Imperial College London via their “Petroleum Engineering & Rock Mechanics Group” webpage^37^. These are mostly images of dry rocks and pore-network models extracted from these rocks for the purpose of pore-scale flow simulations.

The 2D radiograms collected during each injection step are very useful in comparing the flow dynamics at pore-scale for the different injections. In Pak *et al*.^17^ we have used this data to show injection of silica nanoparticles in a carbonate rock can remobilise the trapped oil droplets. In this publication we used a re-sliced form of these 2D radiograms (in the time dimension). Our analysis was, however, limited to qualitative examination of the re-sliced images as the segmentation of these images were challenging. Quantification of the extent of oil remobilisation in this experiment, therefore, remains an open question. New segmentation algorithms may provide reliable segmentation of these data.

Similar to other measurement techniques, μCT imaging is limited by its resolution. Therefore, we believe that acquisition of other independent data such as static and dynamic properties of the rock is useful in understanding the captured images and the improvements offered by the new image processing/analysis algorithms. As such, we refer the readers to our previous publications^14,16,28,29^ in which this rock has been studied from different perspectives and using a range of experimental and modelling techniques.

In summary, the presented data can be used for optimising and testing reconstruction, segmentation, and other image processing algorithms. We foresee potential for these data as a teaching resource as well as a research resource. Currently, no “reference material” exists for μCT, especially within the geosciences context. Given the broad range of independent characterisation of this rock presented in our other publications, we consider this to be a well characterised rock compared to other available datasets.

## Additional information

**How to cite this article**: Pak, T. *et al*. An X-ray computed micro-tomography dataset for oil removal from carbonate porous media. *Sci. Data*. 6:190004 https://doi.org/10.1038/sdata.2019.4 (2019).

**Publisher’s note**: Springer Nature remains neutral with regard to jurisdictional claims in published maps and institutional affiliations.

## Supplementary Material



## Figures and Tables

**Table 1:** Summary of the sample properties.

	Input	Type
1	Rock	Silurian dolomite (Thornton Formation) D = 12.8 mm, L = 34.5 mm
2	Oil	Mineral oil (50%v/v iododecane in dodecane)
3	Nanoparticle	bare silica (US-Nano, 30 nm, product number: US7040)

**Table 2:** Fluid injection/ imaging workflow and conditions for each experiment.

	Injection Step	Flow rate (μL/min)	Pore Volume	Followed by collection of a 3D image labelled as:
1	Initial Water Saturation	500	> 10	Rock-Radiograms-for-3D
2	Oil injection	200 then 500	> 10	Oil-Radiograms-for-3D
3	Water injection: 2D radiograms collected during injection, WI-Radiograms	10	12.25	WI-Radiograms-for-3D
4	Nanofluid (0.06 wt%) injection: 2D radiograms collected during injection, NPI-006-Radiograms	10	3	NPI-006-Radiograms-for-3D
5	Nanofluid (0.12 wt%) injection: 2D radiograms collected during injection, NPI-012-Radiograms	10	3	NPI-012-Radiograms-for-3D

**Table 3:** 3D Images collected at the end of injection steps.

Title	Data Type/File Format	Image Dimensions: 3D images: x,y,z Radiograms: x,y,N (N: number of image)	Overview of the data/Software and algorithm used
Rock-Radiograms-for-3D	Image/RAW 16-bit Unsigned	1024 × 1024 × 2001	Collected at the end of water injection
Rock-Reconstructed	Image/RAW 8 bit	1024 × 1024 × 845	Octopus 8.5^23^, filtered back projection^31^
Rock-Filtered	Image/RAW 8 bit	1024 × 1024 × 845	Avizo^22^, Non-local means^24^
Rock-Segmented-pores	Image/RAW 8 bit	1024 × 1024 × 845	Avizo^22^, Watershed^26,32^
Rock-Mask	Image/RAW 8 bit	1024 × 1024 × 845	Avizo^22^, Watershed^26,32^
Oil-Radiograms-for-3D	Image/RAW 16-bit Unsigned	1024 × 1024 × 2001	Collected at the end of water injection
Oil-Reconstructed	Image/RAW 8 bit	1024 × 1024 × 845	Octopus 8.5^23^, filtered back projection^31^
Oil-Filtered	Image/RAW 8 bit	1024 × 1024 × 845	Avizo^22^, Non-local means^24^
Oil-Segmented-oil	Image/RAW 8 bit	1024 × 1024 × 845	Avizo^22^, Thresholding
Oil-Segmented-water	Image/RAW 8 bit	1024 × 1024 × 845	Avizo^22^, Thresholding
WI-Radiograms-for-3D	Image/RAW 16-bit Unsigned	1024 × 1024 × 2001	Collected at the end of water injection
WI-Reconstructed	Image/RAW 8 bit	1024 × 1024 × 845	Octopus 8.5^23^, filtered back projection^31^
WI-Filtered	Image/RAW 8 bit	1024 × 1024 × 845	Avizo^22^, Non-local means^24^
WI-Segmented-oil	Image/RAW 8 bit	1024 × 1024 × 845	Avizo^22^, Thresholding
WI-Segmented-water	Image/RAW 8 bit	1024 × 1024 × 845	Avizo^22^, Thresholding
NPI-006-Radiograms-for-3D	Image/RAW 16-bit Unsigned	1024 × 1024 × 2001	Collected at the end NP injection: 0.06 wt%
NPI-006-Reconstructed	Image/RAW 8 bit	1024 × 1024 × 845	Octopus 8.5^23^, filtered back projection^31^
NPI-006-Filtered	Image/RAW 8 bit	1024 × 1024 × 845	Avizo^22^, Non-local means^24^
NPI-006-Segmented-oil	Image/RAW 8 bit	1024 × 1024 × 845	Avizo^22^, Thresholding
NPI-006-Segmented-water	Image/RAW 8 bit	1024 × 1024 × 845	Avizo^22^, Thresholding
NPI-012-Radiograms-for-3D	Image/RAW 16-bit Unsigned	1024 × 1024 × 2001	Collected at the end NP injection: 0.12 wt%
NPI-012-Reconstructed	Image/RAW 8 bit	1024 × 1024 × 845	Octopus 8.5^23^, filtered back projection^31^
NPI-012-Filtered	Image/RAW 8 bit	1024 × 1024 × 845	Avizo^22^, Non-local means^24^
NPI-012-Segmented-oil	Image/RAW 8 bit	1024 × 1024 × 845	Avizo^22^, Thresholding
NPI-012-Segmented-water	Image/RAW 8 bit	1024 × 1024 × 845	Avizo^22^, Thresholding
The different injection steps are grouped in sections with alternated shading.			

**Table 4:** 2D Radiograms collected during injection steps.

Title	Data Type/File Format	Image Dimensions 3D images: x,y,z Radiograms: x,y,N (N: number of image)	Overview of the data
WI-Radiograms	Image/RAW 16-bit Unsigned	1024 × 1024 × 2558	Collected during water injection
NPI-006-Radiograms	Image/RAW 16-bit Unsigned	1024 × 1024 × 5850	Collected during NP injection: 0.06 wt%
NPI-012-Radiograms	Image/RAW 16-bit Unsigned	1024 × 1024 × 5972	Collected during NP injection: 0.12 wt%

**Table 5:** Dark current and Flat field (offset and gain) images to be used for image reconstructions.

Title	Data Type/File Format	Image Dimensions	Overview of the data
Dark_Current	Image/RAW 16-bit Unsigned	1024 × 1024 × 10	Useful for reconstruction of all 3D images
Flat_Field	Image/RAW 16-bit Unsigned	1024 × 1024 × 10	Useful for reconstruction of all 3D images

**Table 6:** Summary of the output of this experiment.

	Output	Type
1	Radiograms collected during the injection steps	2D Image
2	Radiograms collected at the end of each injection step	2D Image
3	Reconstruction of the images collected at the end of each injection step	3D Image
4	Filtered Images	3D Image
5	Segmented Images (three phases of rock, oil, aqueous)	3D Image
6	Quantitative information extracted from the above-mentioned images	Data
